# Data-Driven Insights into the Association Between Oxidative Stress and Calcium-Regulating Proteins in Cardiovascular Disease

**DOI:** 10.3390/antiox13111420

**Published:** 2024-11-20

**Authors:** Namuna Panday, Dibakar Sigdel, Irsyad Adam, Joseph Ramirez, Aarushi Verma, Anirudh N. Eranki, Wei Wang, Ding Wang, Peipei Ping

**Affiliations:** 1 Department of Physiology, School of Medicine, University of California, Los Angeles, CA 90095, USA; namuna@g.ucla.edu (N.P.); sigdeldkr@gmail.com (D.S.); 2 NHLBI Integrated Cardiovascular Data Science Training Program (iDISCOVER), University of California, Los Angeles, CA 90095, USA; irsyadadam@g.ucla.edu (I.A.); joseramirez7567@g.ucla.edu (J.R.); aarushiverma999@gmail.com (A.V.); anieranki@g.ucla.edu (A.N.E.); 3 Department of Computer Science, University of California, Los Angeles, CA 90095, USA; weiwang@cs.ucla.edu; 4 Department of Computational Medicine, University of California, Los Angeles, CA 90095, USA; 5 Scalable Analytics Institute (ScAi), University of California, Los Angeles, CA 90095, USA; 6 Department of Bioinformatics and Biomedical Informatics, University of California, Los Angeles, CA 90095, USA; 7 Department of Medicine/Cardiology, University of California, Los Angeles, CA 90095, USA

**Keywords:** Ca^2+^-regulating proteins, oxidative stress, reactive oxygen species, cardiovascular diseases, text mining, knowledge graph

## Abstract

A growing body of biomedical literature suggests a bidirectional regulatory relationship between cardiac calcium (Ca^2+^)-regulating proteins and reactive oxygen species (ROS) that is integral to the pathogenesis of various cardiac disorders via oxidative stress (OS) signaling. To address the challenge of finding hidden connections within the growing volume of biomedical research, we developed a data science pipeline for efficient data extraction, transformation, and loading. Employing the CaseOLAP (Context-Aware Semantic Analytic Processing) algorithm, our pipeline quantifies interactions between 128 human cardiomyocyte Ca^2+^-regulating proteins and eight cardiovascular disease (CVD) categories. Our machine-learning analysis of CaseOLAP scores reveals that the molecular interfaces of Ca^2+^-regulating proteins uniquely associate with cardiac arrhythmias and diseases of the cardiac conduction system, distinguishing them from other CVDs. Additionally, a knowledge graph analysis identified 59 of the 128 Ca^2+^-regulating proteins as involved in OS-related cardiac diseases, with cardiomyopathy emerging as the predominant category. By leveraging a link prediction algorithm, our research illuminates the interactions between Ca^2+^-regulating proteins, OS, and CVDs. The insights gained from our study provide a deeper understanding of the molecular interplay between cardiac ROS and Ca^2+^-regulating proteins in the context of CVDs. Such an understanding is essential for the innovation and development of targeted therapeutic strategies.

## 1. Introduction

Calcium (Ca^2+^) plays a pivotal role in various biological systems, serving as an essential messenger in numerous cellular processes [1,2,3,4,5]. One such critical system is the cardiovascular system, where a network of Ca^2+^-regulating proteins is involved for cardiac functionality. Proteins such as L-type calcium channel (LTCC); ryanodine receptor 2 (RyR2); and troponin C—a myofilament protein—engage in a tightly coordinated series of steps that govern Ca^2+^ dynamics [6,7,8,9,10,11]. These dynamics are crucial for the excitation–contraction cycle (ECC), the process that allows the heart to contract and relax efficiently. In addition to their role in the ECC, Ca^2+^-regulating proteins also have vital roles in cellular energetics. Mitochondrial Ca^2+^-regulating proteins—including the mitochondrial Ca^2+^ uniporter (MCU), mitochondrial sodium/calcium exchanger protein (NCLX), and mitochondrial calcium uptake 1 and 2 (MCU1 and MCU2)—help maintain Ca^2+^ homeostasis in the mitochondria. This is essential not only for normal Adenosine Triphosphate (ATP) generation but also for the regulation of reactive oxygen species (ROS), highlighting the interconnectedness of Ca^2+^-regulatory protein networks in various biomolecular processes [12,13,14].

The intricate relationship between Ca^2+^ and ROS is worth noting. These two agents mutually regulate one another in cellular dynamics. Elevated Ca^2+^ concentrations within a cell can lead to an overproduction of ROS, inducing oxidative stress. This stress is essentially a disruptive imbalance between oxidants, such as ROS, and reductants like antioxidants. This abnormal surge in ROS levels is notorious for causing damage to proteins, lipids, and DNA—a chain of events that ultimately causes cell death [15,16,17,18,19,20,21]. Conversely, an abundance of ROS, and by extension OS, can have profound impacts on cellular Ca^2+^ dynamics. This is evident as OS can remodel Ca^2+^-regulating ion channels, disrupt associated pathways, and alter the functions of regulatory proteins [22,23,24]. Such perturbations in Ca^2+^ dynamics are linked to CVDs like arrhythmia, heart failure, and contractile dysfunction [22,25,26,27]. Given the significant overlap in the literature about Ca^2+^-OS dynamics and CVDs, it becomes vital to systematically explore these interconnections.

To address this need, we utilized advanced text-mining pipelines, specifically employing the CaseOLAP pipeline [28,29]. This tool allowed us to categorize publications into eight distinct CVD categories and score the relevance of 128 cardiac Ca^2+^-regulating proteins within these categories. The derived scores were based on two critical components: “popularity”, measuring the frequency of a given protein in one category versus others in the same category; and “distinctiveness”, assessing the frequency of the target protein in one category compared to alternate categories. Impressively, these scores remain robust even among class imbalances, like varied document counts in CVD categories. A higher score signifies a stronger protein–disease association.

Furthering our analytical endeavors, we formulated a knowledge graph (KG) [30,31]. This KG integrated the scored protein–disease associations with related datasets, including protein information; CVD and OS MeSH descriptors; reference articles; and molecular pathways. The KG analysis provided a comprehensive view, highlighting associations between cardiac Ca^2+^-regulating proteins, CVDs, and OS signaling networks. By revealing these intricate relationships, we aspire that our comprehensive approach will pave the way for future research, potentially guiding the discovery of novel drug targets and therapeutic avenues for CVDs.

## 2. Materials and Methods

This study is designed to elucidate the shared molecular mechanisms between Ca^2+^-regulating proteins and oxidative stress molecules in relation to eight CVD categories, as outlined in Table 1. To achieve this objective, we have established a comprehensive platform that integrates text mining with knowledge graph (KG) analysis.

### 2.1. Data Collection

#### 2.1.1. Document Collection

Initially, we curated a comprehensive corpus of biomedical literature until October 2023, focusing on eight CVD categories and their association with oxidative stress. A total of 1,197,530 unique documents were identified that explored CVDs, with 102,807 of these also addressing oxidative stress (refer to Table 1). The selection of documents was guided by relevant CVD and OS Medical Subject Heading (MeSH) descriptors, which are systematically arranged in a hierarchical framework [32]. This structure facilitated the retrieval of publications across various levels of specificity concerning the CVD categories and oxidative stress.

#### 2.1.2. CVD-MeSH Collection

In this study, we employed eight predefined categories of CVDs, as outlined in Table 1 [33,34]. We compiled associated MeSHs for these CVD categories, including both root nodes and their descendant entries, from the MeSH Library of the National Library of Medicine (NLM) database on 12 June 2023. This comprehensive retrieval resulted in a total of 176 unique CVD-MeSH descriptors. These descriptors are detailed in Supplementary Materials Table S1. The tree structure visualization is available through this link (https://caseolap.github.io/IonChannel/plots/CVD_MeSH_tree.html).

#### 2.1.3. Oxidative Stress (OS)-MeSH Collection

Oxidative stress (OS) is an imbalance between oxidants, such as ROS, and reductants, such as antioxidants, leading to an abnormal increase in ROS levels. In this study, we manually collected OS-relevant molecules from the literature and mapped them to their corresponding MeSH descriptors using the NLM MeSH Library. Mapping of OS-relevant molecules and molecular events into specific, text-mining-applicable MeSH descriptors enhances the precision of text-mining efforts by structuring OS-related data in a way that captures meaningful molecular interactions.

We utilized a total of 75 OS-MeSH descriptors, which are grouped into three primary phases: Initiation of OS (IOS), Modulation of OS (MOS), and Outcome of OS (OOS). This structured categorization represents distinct phases in the OS pathway, allowing for better alignment with specific cardiovascular disease (CVD) associations. Each category is briefly described in the following sections. A detailed classification of OS-MeSH descriptors is available in Supplementary Materials Table S2 and also presented as a tree structure visualization. The tree structure visualization is available through this link (https://caseolap.github.io/IonChannel/plots/oxidative-stress.html).

##### Initiation of OS (IOS)

IOS includes all OS events involved in producing free radical and non-radical species. This phase incorporates three subcategories of chemically reactive species: ROS, which contain oxygen; reactive nitrogen species (RNS), which are derived from nitric oxide; and reactive aldehydes (RA), which are organic compounds with a carbonyl functional group. IOS includes 12 MeSH descriptors that represent a group of small molecules (e.g., radicals).

##### Modulation of OS (MOS)

MOS includes events involving the OS process and progression. This phase incorporates four subcategories: redox metabolites, which result from oxidation/reduction reactions removing oxidative radicals; antioxidants, which contain compounds that inhibit or eliminate oxidation and free radical release; antioxidant enzymes, which contain enzymes that catalyze free radical decomposition; and redox regulating proteins, which contain proteins involved in redox signaling. MOS includes 59 MeSH descriptors that represent a group of carefully curated metabolic molecules, such as redox-regulating cofactors (e.g., glutathione and NAD+/NADH), antioxidant enzymes (e.g., glutathione S-transferases (GSTs)), and other cellularly coexisting antioxidants (e.g., vitamins A, C, and E).

##### Outcome of OS (OOS)

OOS includes OS events involved in the downstream consequences and products of OS. This phase incorporates four subcategories: protein oxidation, which contains protein products following reactions with ROS; lipid peroxidation products, which contain biochemical products of lipid oxidation; oxidative DNA damage, which contains oxidative lesions in DNA; and nitrative DNA damage, which contains nitrative lesions in DNA. OOS includes 6 MeSH descriptors that represent the most well-studied oxidative stress products (e.g., reactive aldehyde 4-HNE and 4-HNE adducts on proteins) and OS-related events (e.g., protein carbonylation and hydroxylation).

#### 2.1.4. Assembly of Cardiac Ca^2+^-Regulating Protein List

Initially, we undertook an advanced search on UniProt using targeted keywords like “Ca^2+^ ion channels”, “heart”, and “human”. This search yielded 105 reviewed proteins linked to cardiac Ca^2+^ dynamics. Further, manual curation was conducted by our team to focus exclusively on proteins that hold functional significance and play a direct, pivotal role in ensuring Ca^2+^ homeostasis in the cardiac cell based on the GO terms, cardiac proteome data, and relevant literature search [35]. After rigorous curation, such as excluding certain tissue-specific proteins, we compiled 128 Ca^2+^-regulating proteins, each associated with a distinct UniProt ID. The comprehensive classification of these proteins is presented in a tabulated format in Supplementary Materials Table S3, detailing protein names, alongside their UniProt IDs. The tree structure visualization is available through this link (https://caseolap.github.io/IonChannel/plots/Ion-channels.html).

#### 2.1.5. Pathway Collection

All pathways associated with the 128 Ca^2+^-regulating proteins were extracted from Reactome knowledgebase.

### 2.2. Workflow Design

**Figure 1:**
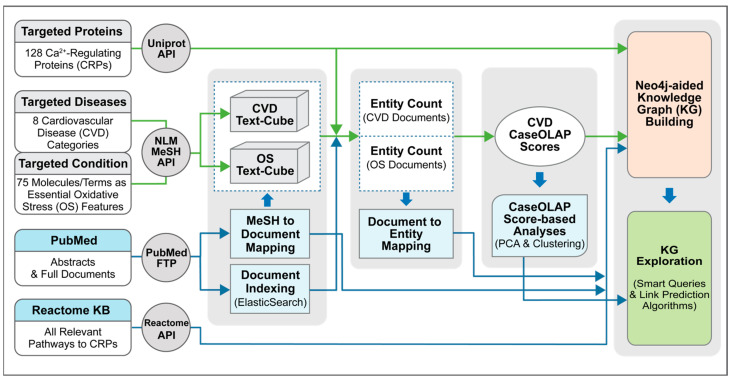
Overview of the workflow. This workflow illustrates the process of extracting, transforming, loading, and analyzing the relevant data sources. The leftmost column represents the data sources: UniProt for the Ca^2+^-regulating proteins, MeSH for oxidative stress (OS) concepts and 8 categories of cardiovascular diseases (CVDs), PubMed (https://pubmed.ncbi.nlm.nih.gov/) for documents, and Reactome (https://reactome.org/) for relevant pathways. Two text cubes were assembled for collections of relevant documents studying OS and CVDs respectively. CaseOLAP scores were computed to quantify the relevance with respect to the CVD categories for every relevant protein. This information was integrated via the construction of a knowledge graph, along with documents, MeSH descriptors, and pathways.

We implemented a text-mining and knowledge-graph platform to better understand the underlying molecular mechanisms of Ca^2+^-regulating proteins involved in 8 CVDs and OS. We executed the CaseOLAP score to reveal the association between Ca^2+^-regulating proteins and 8 CVD categories. We analyzed those protein–disease association scores with unsupervised machine-learning techniques (PCA and hierarchical clustering) [36,37] to gain further insight into the shared roles of proteins. We then constructed a KG graph by incorporating scored proteins, their pathways, CVD and OS MeSH descriptors, and PubMed documents related to CVDs and OS. The workflow (Figure 1) illustrates this process. We further explored the knowledge graph using smart queries and a link prediction algorithm to reveal and propose hidden relationships between the Ca^2+^-regulating proteins, OS, and CVDs.

### 2.3. Knowledge Graph Construction

**Figure 2:**
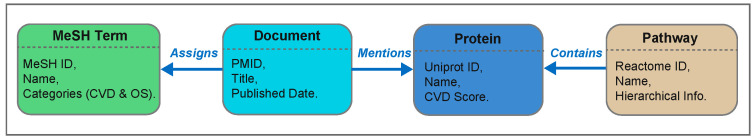
Knowledge-graph data schema. This data schema represents the knowledge graph (KG)’s structure, its node, and edge types. There are 4 nodes (MeSH, document, protein, and pathway), as well as 3 edges (document–assigns–MeSH, document–mentions–protein, and pathway–contains–protein).

We constructed a heterogeneous KG from our protein–disease association scores and other biomedical data sources (UniProt, PubMed, MeSH, and Reactome). Our KG included four node types (protein, document, MeSH, and pathway) and three edge types (document–assigns–MeSH, document–mentions–protein, and pathway–contains–protein). Figure 2 shows the KG schema, and Table 2 and Table 3 list node and edge statistics, respectively.

### 2.4. Software and Tools Utilized for Data Management and Analysis

We employed two widely recognized open-source software tools to manage and analyze our data. The first, Elasticsearch, version 7.14.0, is a powerful indexing, search, and analytics engine renowned for its scalability and speed. It is developed by Elastic and accessible at https://www.elastic.co/. The headquarters of Elastic is located in San Francisco, CA 94108, USA. The second tool used is Neo4j, version 5.18.1, a leading graph database management system known for its efficient management of highly connected data. Further details about Neo4j can be found on its website at https://neo4j.com/, and its offices are based at 400 Concar Dr, San Mateo, CA 94402, USA. These tools were integral to the data handling and analysis phases of our research, providing robust and reliable platforms for our computational needs.

## 3. Results

### 3.1. Interaction Among Ca^2+^-Regulating Proteins and both CVD and OS Categories

**Figure 3:**
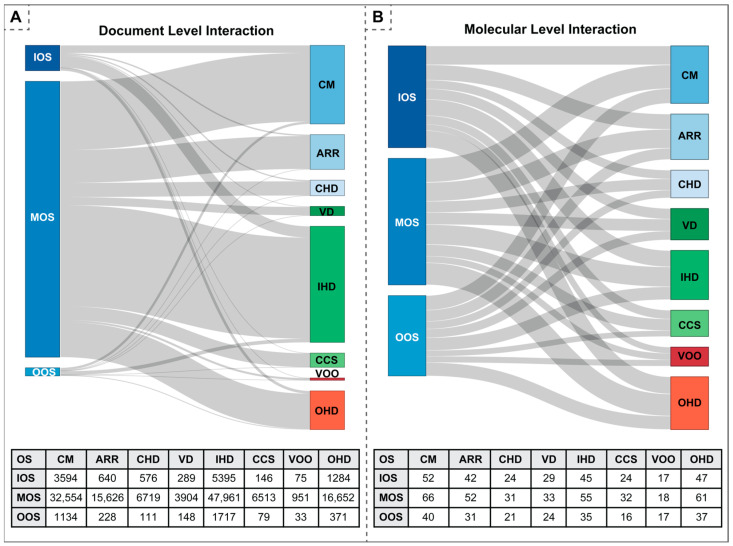
Visualization of document-level association and molecular-level interaction between oxidative stress (OS) and cardiovascular disease (CVD) categories. (**A**) The top panel is a Sankey diagram outlining the document-level OS-CVD association. The column height represents the total number of documents associated with each OS phase and CVD category; the chord thickness is defined by the proportion of documents shared between each OS-CVD pair. An interactive version of this plot is also available online (https://caseolap.github.io/IonChannel/rcodes/docs-intersection.html). The bottom panel shows the detailed document counts for the Sankey diagram above. (**B**) The top panel is a Sankey diagram representing the molecular-level interaction between OS and CVD categories that are specifically relevant to Ca^2+^-regulating proteins. The column height represents the total number of proteins related to each OS phase and CVD category, and the thickness of the chord represents the number of proteins shared among each OS phase and CVD category. The interactive version is available online (https://caseolap.github.io/IonChannel/rcodes/protein-intersection.html). The bottom panel provides the numbers that went into the Sankey diagram above.

We systematically examined the interaction among Ca^2+^-regulating proteins and both CVD and OS categories from a quantitative perspective, as depicted in Figure 3A,B. Specifically, Figure 3A elucidates the number of shared documents across distinct CVD and OS categories, while Figure 3B delineates the overlap of proteins across CVD and OS categories.

#### 3.1.1. Documents in OS-CVD Categories

We collected 102,807 unique documents studying CVDs and OS. A pronounced volume of publications was observed at the nexus of modulation of oxidative stress (MOS) with both ischemic heart disease (IHD) and cardiomyopathy (CM). Although the distribution of publications in pairwise OS-CVD categories was disproportionate, each OS-CVD category contained at least some publications. This encouraged us to explore the protein-level interaction (e.g., proteins behind the OS-CVD associations) by identifying the proteins in each OS-CVD category.

#### 3.1.2. Proteins in OS-CVD Categories

Inspired by the interaction seen at the document level (see Figure 3A), we further quantified protein-level interaction among the CVD and OS categories (see Figure 3B). The strength of the interaction is calculated based on the shared proteins with a non-zero CaseOLAP score. Although the documents were disproportionately represented in the OS-CVD categories, the proteins were more uniformly distributed. This suggests that, despite the disproportionate studies in the OS-CVD categories (as represented by the disproportionate number of publications), we can obtain useful biological information: Ca^2+^-regulating proteins serve biological functions in each stage of OS in each major CVD category.

### 3.2. CaseOLAP Score Analysis

**Figure 4:**
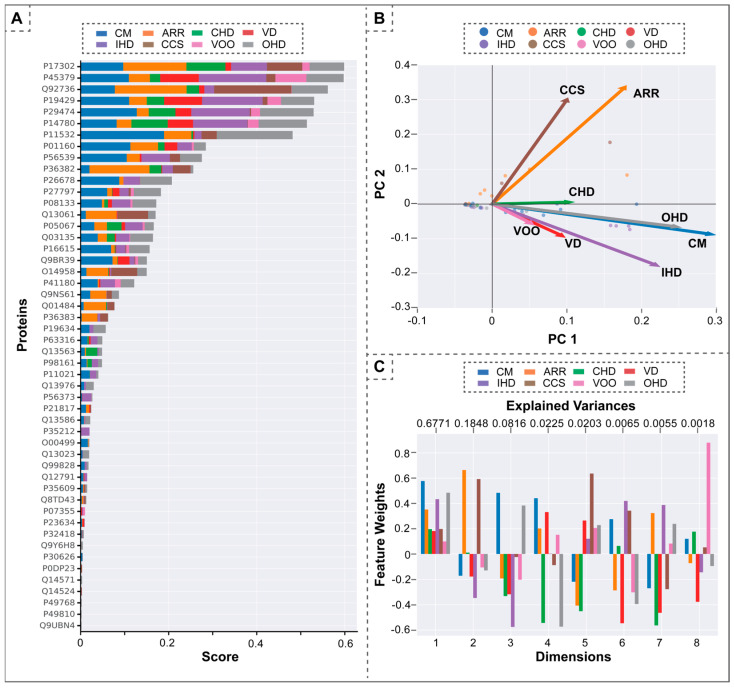
Analysis of top-scoring proteins and PCA results: (**A**) demonstrates the top-scoring proteins across all cardiovascular disease (CVD) categories, visualized as a stacked bar chart; and (**B**) illustrates the results of the protein’s principal component analysis (PCA) within the 8-dimensional vector space corresponding to the CVD categories. This is subsequently projected onto a 2-dimensional plane defined by the primary principal components (PC1 and PC2). Individual dots signify distinct proteins, while the arrow-headed vectors represent the eight CVD categories in relation to PC1 and PC2. This illustration helps in comparing the behavior of the CVD categories based on the scoring proteins, providing insights into potential groupings of proteins operating via analogous molecular mechanisms. Notably, cardiac conduction system disease (CCS) and arrhythmias, cardiac (ARR), emerge as distinct from the remaining CVDs. (**C**) indicates the contribution of each CVD within the PCA dimensions. Considering the first two dimensions (i.e., PC1 and PC2), the first dimension is the positive superposition of all diseases, and ARR and CCS dominate the second dimension. The color bar in (**A**–**C**) represents different CVD categories.

In our analysis of the corpus, a notable 78 out of the 128 identified Ca^2+^-regulating proteins acquired a CaseOLAP score in relation to at least one CVD category. This indicates an association of these proteins with one or more of the eight delineated CVDs. A comprehensive representation of all scoring proteins across CVD categories is illustrated in Figure 4A. The association patterns of proteins within individual CVDs seem heterogeneous; these proteins exhibit varied relationships across the eight CVDs. Notably, cardiomyopathy (CM) is linked with 67 of the 78 scoring proteins, underscoring its significant correlation with Ca^2+^-regulating protein functionality. Similarly, ischemic heart disease (IHD) and arrhythmias (ARR) have associations with 55 and 52 proteins, respectively. By utilizing CaseOLAP scores, we further implemented dimensionality reduction and clustering methods to discern the scoring patterns of these proteins.

#### 3.2.1. Principal Component Analysis (PCA)

PCA is a machine-learning technique for dimensionality reduction [37]. We utilized PCA to transform the eight-dimensional protein score vectors into a more understandable two-dimensional space, as depicted in Figure 4B. Within the PCA plot, each dot symbolizes a distinct protein, whereas each arrowhead vector provides a 2D projection corresponding to a specific CVD category. A notable observation from this analysis is the distinct positioning of two CVDs, ARR and CCS, which are differentiated from the other six categories based on their respective CaseOLAP scores. Figure 4C showcases the factor loadings of the principal components. PC1 comprises a relatively even combination of all CVDs, while PC2 is dominated by CCS and ARR.

#### 3.2.2. Clustering Behavior of Ca^2+^-Regulating Proteins

**Figure 5:**
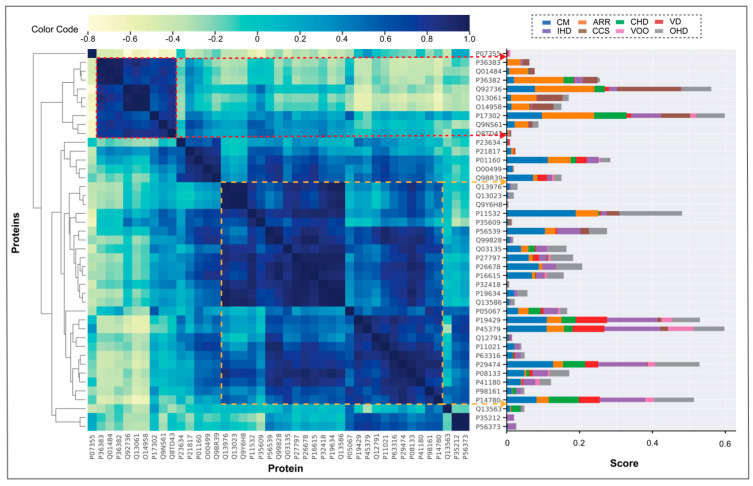
Clustering behaviors of CVD proteins. The cluster image demonstrates the hierarchical clustering of protein scores into a two-dimensional cluster plot. The cluster is formed based on the Euclidean distance metric in 8-dimensional protein vector space. The distance scores obtained are distributed in a cluster plot ranging from −0.8 to 1.0, as shown in the color legend. A darker intensity indicates closely clustered proteins. The results demonstrated two significant clusters. The cluster enclosed with a red square represents a group of proteins associated with arrhythmias, cardiac (ARR), and cardiac conduction system disease (CCS). The cluster enclosed with a yellow square represents a group of proteins related to cardiomyopathies and heart failure (CM), myocardial ischemia (IHD), and other heart diseases (OHDs). The bar plot on the right is the visualization of the CaseOLAP scores of proteins rearranged based on the clustering.

Using an Euclidean distance metric, we applied hierarchical clustering [36] to the proteins under study based on their eight-dimensional CaseOLAP scores across the eight CVD categories. This clustering technique groups proteins with similar scores, reflecting their potential biomedical relevance within specific CVDs. Our analysis recognized distinct protein clusters corresponding to specific CVDs based on their CaseOLAP scores, as shown in Figure 5. For instance, proteins enclosed within the red boundary predominantly influence the ARR and CCS categories in contrast to other CVDs. Similarly, a cluster of proteins enclosed within the yellow square shows a pronounced association with CM, IHD, and CCS. These insights guided us for the subsequent knowledge-graph analyses.

### 3.3. KG Analyses

We analyzed the KG using cypher queries (cypher query information can also be found on the Neo4j webpage) and a link prediction algorithm.

#### 3.3.1. KG Analysis: Queries

Implementing queries, we searched for the Ca^2+^-regulating proteins in the document corpus mentioned, together with OS molecules. Our findings reveal that 59 out of the 128 examined Ca^2+^-regulating proteins demonstrate associations with at least one oxidative stress molecule and a CVD. Notably, these 59 proteins display a diverse range of CaseOLAP scores across the different categories of cardiovascular diseases. Of particular interest, 55 of these proteins are involved in cardiomyopathy, the most prevalent disease category linked to calcium regulation and oxidative stress interplay. Ischemic heart disease follows closely, with 50 proteins demonstrating associations, underscoring it as the second most prevalent condition in this context. We have provided a comprehensive breakdown of the interconnections between Ca^2+^-regulating proteins and oxidative stress molecules across each cardiovascular disease category in Supplementary Table S4.

To further dissect the molecular mechanism of these associations, we delved into our knowledge graph, employing cypher queries to elucidate shared oxidative stress molecules and pathways that cluster within the ARR and CCS disease categories. The following subsections present the most relevant OS molecules and pathways corresponding to those proteins.

##### Significant OS Molecules in ARR and CCS Cluster

In our analysis, we have identified a pivotal interplay between specific OS molecules and Ca^2+^-regulating proteins within the cluster of ARR and CCS diseases. Utilizing cypher queries within our knowledge graph, we systematically isolated proteins that co-occur with OS molecules within the same literature sources. This allowed us to calculate an average CaseOLAP score for these OS molecules across each CVD category, as defined by Equation (1):

OS molecule score = mean (protein’s CaseOLAP score in each CVD category)(1)

**Figure 6:**
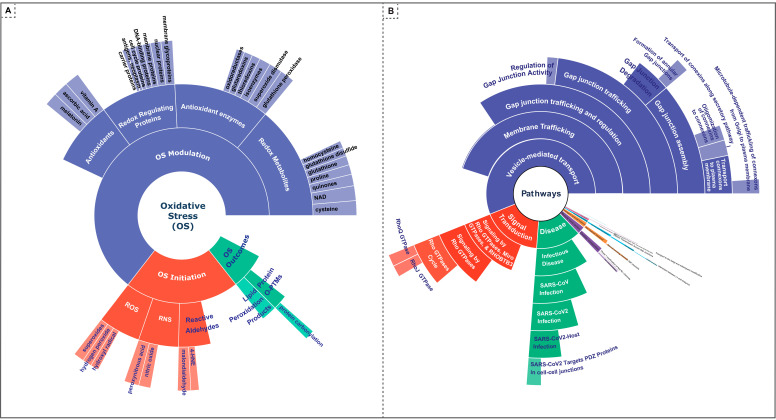
Significant OS molecules and pathways in an ARR-CCS cluster: This figure presents sunburst visualizations illustrating significant oxidative stress (OS) molecules (**A**) and pathways (**B**) linked to the proteins identified in the arrhythmias, cardiac (ARR), and cardiac conduction system disease (CCS) clusters. Quantitative assessments of these OS molecules and pathways were conducted by calculating their respective scores based on the CaseOLAP scores of proteins within each cardiovascular disease (CVD) category. For OS molecules, scores were determined using the formula inside the parenthesis (OS molecule score = mean (protein’s CaseOLAP score in each CVD category)). For pathways, the score was computed utilizing the formula inside the parenthesis (pathway score = mean (protein’s score in each CVD category ∗ (1 − *p*-value)). In both (**A**,**B**), the arc thickness symbolizes the cumulative score for each molecule and pathway across the eight CVD categories. The outermost ring of the sunburst represents individual OS molecules (**A**) and pathways (**B**), with the arc width reflecting the score magnitude. The successive inner rings are organized hierarchically, with (**A**) categorizing candidate molecules and (**B**) sorting pathways based on their classification. An interactive version of these visualizations is available at https://caseolap.github.io/IonChannel/plots/os.html and https://caseolap.github.io/IonChannel/plots/os-cvd-pathways.html. For a more detailed and enhanced examination, you may zoom in on these interactive figures via the provided links.

The findings are visually represented through an interactive sunburst chart (Figure 6A), which displays all the significant OS molecules in an ARR-CCS cluster based on the hierarchical relationship of OS molecules through the series of circles moving outwards according to their hierarchy. The inner, middle, and outer circles represent the category, subcategory, and individual molecules, respectively. The significant OS molecules are at the outermost circumference, where the thickness of the arc represents their collective scores in ARR and CCS categories. Circumference of concentric inner cords is calculated based on the total score of their descendants.

Our analysis shows that from a total of 75 OS molecules analyzed, 31 exhibit a significant association with the proteins under study. Dissected by OS phase, 5 molecules belong to the initiation phase, 23 to the modulation phase, and 3 to the outcome phase. All of those OS molecules are provided in Supplementary Table S5.

Highlighted OS molecules in our study, such as hydroxyl radicals, superoxides, and hydrogen peroxide, have been found in increased amounts in failing myocardium [38,39,40]. Experimental evidence has shown that an increased concentration of these ROS molecules causes calcium overload in the cardiomyocytes by modulating the properties of Ca^2+^-regulating channels, ultimately causing contractile dysfunction and arrhythmias [40,41]. For example, exposure to hydroxyl radicals causes Ca^2+^ overload in the cardiomyocytes by increasing the open probability of cardiac ryanodine receptors, which control Ca^2+^ release from the sarcoplasmic reticulum to the cytoplasm [42,43]. Increased calcium influx through voltage-gated calcium channels is observed experimentally by brief exposure to hydrogen peroxide in ventricular myocytes [44,45].

Conversely, the antioxidants highlighted by our results (e.g., glutathione, glutathione peroxidase, superoxide dismutase, thioredoxins, vitamin A, and ascorbic acid) [46,47] are essential for cardiovascular health [46]. Cells synthesize antioxidant compounds and enzymes to maintain redox homeostasis and mitigate ROS-induced damage. For example, superoxide dismutase utilizes superoxide to generate hydrogen peroxide, which catalase further metabolizes to water and oxygen [48].

##### Significant Pathways in an ARR and CCS Cluster

To elucidate the molecular mechanisms of protein clusters within the ARR and CCS categories, we collected associated molecular pathways using queries in KG. We scored relevant molecular pathways by using Equation (2), which calculates the pathway score by integrating both the protein associations and the significance levels (*p*-values) of the pathways.

Pathway score = mean (protein’s score in each CVD category ∗ (1 − *p*-value)) (2)

Initially, we collected all associated pathways tied to each protein. Since multiple proteins are associated with a single pathway, we utilized reverse mapping and collected all the proteins involved in the specific pathways. Next, we incorporated the *p*-value of the pathway into the CaseOLAP score of associated proteins. We further took their means to create the final score for a pathway.

All the significant pathways in an ARR and CCS cluster are visualized in the interactive sunburst visualization (Figure 6B) following the hierarchical relationship of the pathways through the series of circles moving outwards according to their hierarchy. The pathway hierarchy is based on the Reactome knowledgebase. The significant pathways are distributed across the circles based on their hierarchy, where the thickness of the arc represents their collective scores in ARR and CCS, added to the total score of their descendants. There is a total of 50 significant pathways, and they are also provided in table format in Supplementary Table S6.

Some notable pathways highlighted by our exploration are gap junction trafficking and regulation and their descendant pathways. The descendant pathways include gap junction assembly, gap junction degradation, oligomerization of connexins into connexons, transport of connexins along the secretory pathway, transport of connexons to the plasma membrane, microtubule-dependent trafficking of connexons from Golgi to the plasma membrane, and so on.

Gap junctions are clusters of intercellular channels connecting neighboring cells, facilitating the direct exchange of ions and small molecules. They are composed of connexins (six transmembrane protein units) that are transported to the plasma membrane after oligomerizing into hexameric assemblies called connexons. The activity of these intercellular channels is regulated, particularly by intramolecular modifications such as phosphorylation, which appears to regulate connexin turnover, gap junction assembly, and the opening and closure (gating) of gap junction channels. Excessive OS leads to reduced gap junction protein connexin (Cx43), a protein critical for normal cardiac conduction function. Reduced connexin levels may slow conduction and facilitate the proarrhythmic mechanism [49,50,51].

#### 3.3.2. KG Analysis: Link Prediction Algorithm

**Figure 7:**
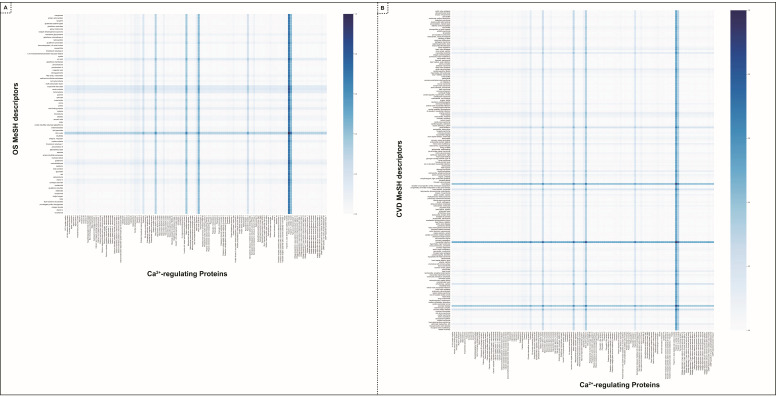
Link prediction analysis implementing graph algorithm: The heatmap depicts the predictive association scores derived from link prediction algorithms between oxidative stress (OS) molecules and Ca^2+^-regulating proteins (**A**), as well as between Ca^2+^-regulating proteins and cardiovascular diseases (CVDs) (**B**). The color intensity within the heatmap corresponds to the strength of the predicted association, with darker shades indicating a higher likelihood of connection between entities. Notably, proteins such as cardiac troponin I, nitric oxide synthase, matrix metalloproteinase-9, and gap junction alpha-1 protein, which play a role in calcium regulation, are identified with a high degree of probability as being associated with both OS molecules and CVD, underscoring their potential relevance in the underlying molecular mechanisms.

We applied a link prediction algorithm to identify new biomedical knowledge from the patterns in the data [52]. In our case, we used link prediction to propose undiscovered relationships between (i) Ca^2+^-regulating proteins and OS molecules and (ii) Ca^2+^-regulating proteins and CVDs (Figure 7). It was implemented on our KG-graph data from Neo4j’s Graph Data Science Library. The algorithm uses a graph’s topology to compute scores based on the closeness of a pair of nodes. These scores are then used to predict missing relationships. A higher score suggests a higher likelihood of an undiscovered node–node relationship.

##### Link Prediction Between Ca^2+^-Regulating Proteins and OS-MeSH Descriptors

The link prediction algorithm ranked possible protein–OS relationships, highlighting significant proteins–OS pairs. The top predicted protein–OS pair was cardiac troponin I, and nitric oxide. In fact, it predicts that troponin I links with all OS molecules and that nitric oxide links with all proteins with a significant link prediction score (see Figure 7A). Both are essential and responsible for the heart’s excitation–contraction mechanism. Cardiac troponins are Ca^2+^-regulatory proteins accountable for the heart’s excitation–contraction mechanism [53,54,55]. NO influences the Ca^2+^ channels, facilitates the cGMP and PKG-dependent phosphorylation of troponin I, and attenuates myofilament response to calcium. Moreover, nitric oxide produced by endothelial cells regulates the excitation–contraction cycle of the heart by promoting vascular relaxation [56,57,58,59].

The other significant proteins were Matrix metalloproteinase-9, Nitric oxide synthase, endothelial, and gap junction alpha-1 protein, and their association with Nitric Oxide is significantly higher than other OS-MeSH descriptors. The table of the link prediction score is also provided in Supplementary Table S7.

##### Link Prediction Between Ca^2+^-Regulating Proteins and CVD-MeSH Descriptors

The link prediction analysis between proteins and CVD-MeSH descriptors predicted the notable protein–CVD pair. The top predicted protein–CVD pair was myocardial infarction and troponin I, indicating a strong possible connection between them (see Figure 7B). The other top four proteins associated with myocardial infarction with higher link prediction scores were nitric oxide synthase, endothelial, matrix metalloproteinase-9, gap junction alpha-1 protein, and ryanodine receptor 2. These proteins were also strongly associated with the OS-MeSH descriptors (see Figure 7A), such as nitric oxide, carrier proteins, superoxide dismutase, and other CVD-MeSH descriptors specifying the interconnection between the proteins, OS, and CVDs. For example, our results highlighted a triangular association between myocardial infarction, troponin I, and nitric oxide. The table of the link prediction score is also provided in Supplementary Table S8.

## 4. Discussion

The findings of this study reveal significant correlations between cardiac Ca^2+^-regulating proteins and OS in the pathogenesis of various cardiovascular diseases. This interaction is relevant given the established role of ROS in disrupting Ca^2+^ homeostasis, which can lead to functional and structural changes in essential proteins involved in cardiac excitation–contraction coupling. The insights gained here underscore the complex molecular interplay that fosters disease progression in CVD, opening avenues for targeted therapeutic intervention.

Our study adopts a novel, granular approach by using specific molecular entities and their MeSH-mapped descriptors to define oxidative stress at a molecular level rather than relying on broad, oversimplified OS concepts. This strategy allows for a more precise characterization of ROS, making it possible to text-mine oxidative stress in a nuanced manner that captures detailed molecular interactions. By mapping oxidative stress in this way, we can extract associations at a more tangible molecular level, thereby improving the specificity of text-mining results and enhancing the biological relevance of our findings.

Our results highlight the detrimental impact of ROS, such as hydroxyl radicals, superoxide, and hydrogen peroxide, on Ca^2+^-regulating proteins, with pronounced associations in multiple CVD categories, notably arrhythmias, cardiac conduction system diseases, and cardiomyopathy. Elevated ROS levels induce Ca^2+^ overload by altering Ca^2+^-regulating channels and ion transport mechanisms, establishing a self-sustaining feedback loop that exacerbates oxidative stress and fosters a pathological environment. An example of this is the OS molecules highlighted by our study, such as hydrogen peroxide, whose exposure increases the open probability of cardiac ryanodine receptors, leading to excessive Ca^2+^ release from the sarcoplasmic reticulum, a mechanism associated with arrhythmias and functional degradation in patients with impaired cardiac conduction. A critical protein in this context is the cardiac ryanodine receptor RyR2, which is predominantly expressed in cardiac muscle and plays a central role in cardiac Ca^2+^ signaling.

The KG analysis further elucidates shared molecular pathways and associations between Ca^2+^-regulating proteins and oxidative stress across eight CVD categories. Distinct protein clusters, particularly those associated with cardiac arrhythmias and conduction disorders, reveal a specific vulnerability to ROS-mediated damage. These proteins are often linked with ROS-related pathways, such as gap junction trafficking and redox signaling, pointing to an integrated role of OS within cardiac signaling networks. For example, gap junctions, particularly those involving connexin 43 (Cx43), are critical for electrical conduction in the heart. OS-induced reduction of Cx43 impairs intercellular communication, a mechanism linked to increased arrhythmogenic risk, demonstrating how OS exacerbates conduction abnormalities.

A key aspect of the link prediction analysis is the identification of potential novel associations between Ca^2+^-regulating proteins and oxidative stress molecules, with particular emphasis on nitric oxide (NO) and cardiac troponins. Nitric oxide, primarily synthesized by nitric oxide synthase 3 (NOS3, also known as endothelial nitric oxide synthase), plays a dual role in cardiovascular health and disease. While NO is essential for maintaining vascular tone and promoting blood flow, excessive NO production during ischemic reperfusion injury contributes to myocardial infarction and cardiac damage. During reperfusion, NOS3 generates high levels of NO that react with superoxide, forming peroxynitrite, a highly reactive molecule known to cause oxidative damage to lipids, proteins, and DNA. This contributes to the pathophysiology of ischemic reperfusion injury, exacerbating myocardial cell death and inflammation. Our analysis shows a strong association of NOS3 with ischemic heart disease, underscoring its potential as both a therapeutic target and a biomarker for myocardial infarction and ischemic damage.

Cardiac troponins, especially the isoforms cardiac troponin I (cTnI) and troponin T (cTnT), are also well-established biomarkers for several CVDs, including myocardial infarction and heart failure. Their increased expression in CVD patients reflects myocardial injury or stress, thus explaining their association with certain CVD categories in our analysis. The interaction between NO and cardiac troponin I, a critical regulatory protein in excitation–contraction coupling, suggests that NO may modulate Ca^2+^ sensitivity, influencing cardiomyocyte contractility. This interaction aligns with previous findings that NO facilitates cGMP-mediated phosphorylation of troponin I, modifying myofilament Ca^2+^ responsiveness. Together, these interactions underscore the complexity of redox signaling in the heart and highlight NOS3 as a critical player in oxidative damage during ischemic reperfusion, suggesting new pathways for redox-based therapeutic strategies in ischemic heart disease.

Applying CaseOLAP scoring allowed for the quantification of protein–disease associations, revealing that cardiomyopathy has the highest incidence of OS-related protein involvement. This is likely due to the chronic nature of cardiomyopathy, where sustained oxidative stress continually remodels Ca^2+^ signaling pathways, leading to progressive cardiac dysfunction. The prevalence of oxidative damage in cardiomyopathy underscores the therapeutic potential of antioxidants in disrupting the OS-Ca^2+^ feedback loop, potentially mitigating disease severity.

In summary, this study employs a text-mining approach combined with a knowledge graph to delineate associations between cardiac Ca^2+^-regulating proteins and oxidative stress across eight categories of CVDs. By integrating fragmented pieces of data pertaining to proteins, OS-MeSH, and CVD-MeSH from an extensive corpus of PubMed articles, we provide new insights into the shared molecular pathways underpinning these associations. This strategy, grounded in the co-occurrence of highly specific molecular entities, synthesizes a vast array of knowledge, enabling the discovery of novel connections. These findings contribute to a deeper understanding of the molecular interplay between Ca^2+^-regulating proteins and oxidative stress, with implications for early detection tools and therapeutic strategies in CVDs.

## 5. Conclusions

Mutual interaction between the Ca^2+^-regulating proteins and OS has been associated with cellular processes in cardiovascular health and disease. In the present study, we investigated the impact of OS on cardiac Ca^2+^-regulating proteins with respect to the eight major CVD categories by utilizing a text-mining algorithm combined with a knowledge graph. The key findings are as follows: (1) Ca^2+^-regulating proteins are distinctly associated with cardiac arrhythmias (ARR) and cardiac conduction system diseases (CCS) compared to other CVD categories. (2) In total, 59 out of the 128 Ca^2+^-regulating proteins are found in OS-associated CVDs. (3) Cardiomyopathy is the highest-occurring disease category associated with the mutual effect of OS and Ca^2+^-regulating proteins. (4) Utilizing a link prediction algorithm, hidden and possible relationships were emphasized among Ca^2+^-regulating proteins, OS molecules, and CVDs. Our informatics study on the participation of Ca^2+^-regulating proteins in OS-associated cardiac diseases should help find novel early-detection tools and therapeutic strategies.

## Figures and Tables

**Table 1:** Document statistics per CVD categories.

CVD Category	Abbreviation	Major Root Nodes (MeSH)	No. of CVD Publications Collected	No. of OS-Related Publications Within CVD
Cardiomyopathies and heart failure	CM	C14.280.238, C14.280.434	247,436	34,063
Arrhythmias, cardiac	ARR	C14.280.067	239,060	15,960
Heart defects, congenital	CHD	C14.280.400	154,992	7183
Heart valve diseases	VD	C14.280.484	137,197	4127
Myocardial ischemia	IHD	C14.280.647	473,233	50,435
Cardiac conduction system disease	CCS	C14.280.123	100,841	6613
Ventricular outflow obstruction	VOO	C14.280.955	42,942	1003
Other heart diseases (cardiomegaly, endocarditis, heart arrest, heart rupture, ventricular dysfunction, heart neoplasms, and pericarditis)	OHD	C14.280.195, C14.280.282, C14.280.383, C14.280.470, C14.280.945, C14.280.459, C14.280.720	215,416	17,295

Table 1 presents document collection statistics in each CVD category and the OS-CVD interface. We collected a total of 1,197,530 unique documents, all studying at least one CVD. Each CVD category is broken down by name, abbreviation, associated MeSH tree numbers, number of documents studying the CVD category, and number of documents also studying OS.

**Table 2:** Node statistics.

Nodes	Total Unique Nodes
Document nodes	1,197,530
MeSH nodes	251 (75 are OS, 176 are CVD)
Protein nodes	128
Pathway nodes	496

The total number of nodes in our knowledge graph is provided in this table.

**Table 3:** Edge statistics.

Edges	Edge Name	Count
Document to protein	Mentions	13,211
Document to MeSH	Assigns	1,698,233
Protein to pathway	Contains	1542

The total number of edges in our knowledge graph is provided in this table.

## Data Availability

The original contributions presented in this study are included in the article/Supplementary Materials. Further inquiries can be directed to the corresponding author(s).

## Supplementary Materials

The following supporting information can be downloaded at https://www.mdpi.com/article/10.3390/antiox13111420/s1, Table S1: CVD-MeSH descriptors; Table S2: Oxidative stress MeSH descriptors; Table S3: Ca^2+^-regulating proteins; Table S4: Ca^2+^-OS-CVD interface; Table S5: OS molecules associated with proteins in ARR and CCS; Table S6: Pathways associated with proteins in ARR and CCS; Table S7: OS–protein link prediction score; Table S8: CVD–protein link prediction score.
